# Surgical management of a foramen magnum tumor via a far-lateral approach using an oblique straight incision: a case series report and technique note

**DOI:** 10.3389/fonc.2024.1391002

**Published:** 2024-06-12

**Authors:** Jie Bai, Zhi-heng Jian, Peng Chen, Ye Cheng, Ya-ming Wang, Gang Chen, Xin-ru Xiao

**Affiliations:** ^1^Department of Neurosurgery, Xuanwu Hospital, Capital Medical University, Beijing, China; ^2^Department of Nenrosurgery, Zhuhai’s People Hospital (Zhuhai Clinical Medical College of Jinan University), Zhuhai, China

**Keywords:** incision, microsurgery, complication, far lateral approach, suboccipital triangle

## Abstract

**Objective:**

To review our single-institution experience in the surgical management of foramen magnum tumors via a far-lateral approach using an oblique straight incision.

**Methods:**

From October 2023 to January 2024, four cases of tumors in the foramen magnum area treated at the Capital Medical University-affiliated XuanWu hospital neurosurgery department were involved in this study. All cases were managed with a far-lateral approach using an oblique straight incision. We retrospectively reviewed the clinical and imaging data, as well as the surgical strategies employed.

**Results:**

Three cases of foramen magnum meningiomas and one case of glioma of the ventral medulla. All cases underwent a far-lateral approach using an oblique straight incision; all cases had a gross total resection, and the wounds healed well without cerebral fluid leakage or scalp hydrops. Except for one case of right foramen magnum meningioma, which had dysphagia and pneumothorax, the other cases were without any postoperative complications.

**Conclusion:**

A far-lateral approach using an oblique straight incision can preserve muscle integrity and minimize subcutaneous exposure, allowing for complete anatomical reduction of muscles. This craniectomy method is simple and replicable, making it worthy of further clinical practice.

## 1 Introduction

Although the morbidity of tumors in the foramen magnum area is low (1–3), surgical treatment of tumors in this area is still a major challenge due to the deep and narrow surgical field. A far-lateral approach is the most common approach for dealing with lesions in this area (4–7). Until recently, the high complication rate was still concerning among neurosurgeons, especially involving cerebral fluid leakage and scalp hydrops (8). Some researchers attribute CSF leakage to the inadequate suture of muscles (9). Hence, ensuring the integrity of muscle structure during craniectomy is of utmost importance to prevent CSF leakage and scalp hydrops. A straight incision applied in a far-lateral approach has been reported, but the clinical application value is still not explicit (10). In this study, we applied an oblique straight incision in four cases of tumor in the foramen magnum area using a far-lateral approach to evaluate its application value.

## 2 Clinical materials and methods

### 2.1 Patient cohort

Four cases of tumors in the foramen magnum were surgically treated from October 2023 to January 2024. The age, sex, clinical presentation, radiological findings, tumor location, surgical details, pathology, postoperative complications, surgical outcome, and follow-up data of the patients were collected (**Table 1**).

**Table 1:** Summary of cases, clinical features, and surgical management outcomes.

Case No	Sex	Age	Side	Tumor position	Preoperative neurological deficit	Pathology	Complication	Approach/exposure range	Intraoperative findings	Follow-up information
1	Female	35	right	Outramedullar, jugular foramen invaded	III grade of both limbs for muscle strength, Babinski sign (+) (both sides)	meningioma	Dysphagia and pneumothorax	paracondylar	Tumor texture: hard; boundary clear. Tumor grows into jugular foramen. Tumor and glossopharyngeal nerve with tight adhesion.	Postoperatively, after 6 months, the IV grade of both limbs for muscle strength and swallowing function recovered.
2	Male	55	right	Outramedullar, lower clival region	negative	meningioma	None	supracondylar	Tumor texture: soft; boundary is clear.	Postoperatively, after 3 months, there was no newly developed neurological deficit.
3	Female	69	central	Outramedullar, lower clival region	negative	meningioma	None	transcondylar	Tumor texture: soft; boundary is clear.	Postoperatively, after 3 months, there was no newly developed neurological deficit.
4	Male	34	left	Intramedullar, left medulla oblongata	Left upper and lower limbs, III grade, Babinski sign (+) (left)	glioma	None	paracondylar	The tumor was an intramedullary growth. Tumor texture: hard. The boundary is not clear. Guided by IONM	Postoperatively, after 1 month. The left upper and lower limbs remained in III grade; there was no newly developed neurological deficit.

IONM, intraoperative neurophysiological monitoring.

### 2.2 Surgical position, incision design, and surgical treatment

**Figure 1:**
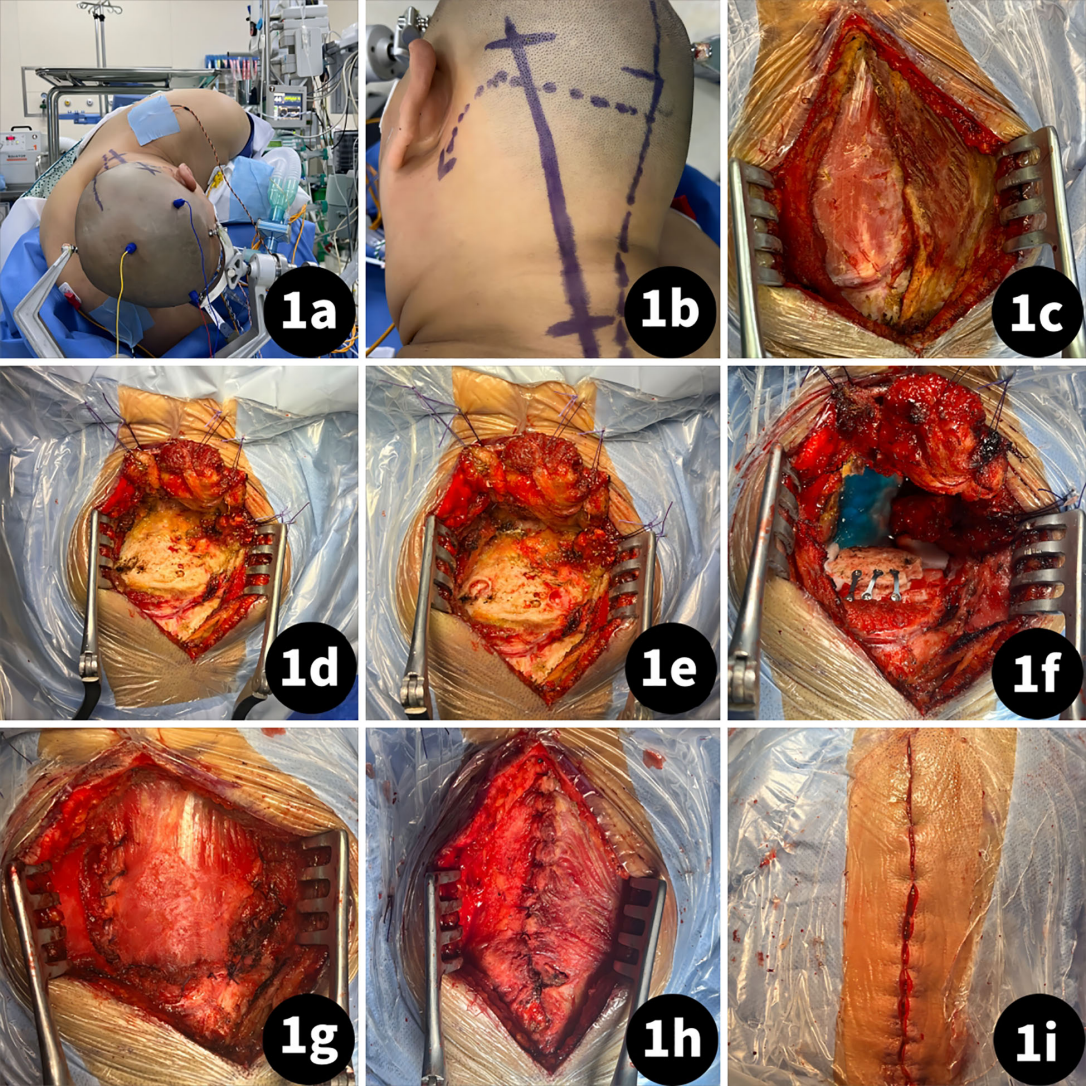
Craniectomy procedure of a far-lateral approach using an oblique straight incision. **(A)** Patient was placed in lateral position; **(B)** Incision designed as a straight oblique incision within the hairline behind the ear, with the lower boundary reaching the C4 spinous process of the midline and the upper boundary reaching 1 cm above the level of external occipital protuberance; **(C)** Stretch the splenius capitis muscle follow the fiber; **(D)** Cut the semispinalis capitis muscle from the upper margin and turn to Lower edge of incision; **(E)** peel the rectus capitis posterior major muscle downward to expose the bony landmarks; **(F)** bone flap retained after tumor resection and dura suture; **(G)** semispinalis capitis muscle suture with fully anatomical reduction; **(H)** splenius capitis muscle suture with fully anatomical reduction; **(I)** scalp suture.

All cases were performed by a senior neurosurgeon. Each patient was placed in a 3/4 prone position with their head slightly turned to the opposite side, their faces slightly rotated to the ventral side, and their neck slightly bent to ensure the lateral external auditory canal was at the highest point. The shoulder was pulled downward, and fixation was achieved using the Mayfield head frame (**Figure 1A**). The incision was a straight oblique one within the hairline behind the ear, with the lower boundary reaching the C4 spinous process of the midline and the upper boundary reaching 1 cm above the level of the external occipital protuberance (**Figure 1B**). The splenius capitis muscle was stretched along its fibers without cutting it up (**Figure 1C**). The semispinalis capitis muscle was cut from the upper margin and turned towards the lower edge of the incision (**Figure 1D**). Care was taken to identify the suboccipital triangle and vertebral artery during this process. The rectus capitis posterior major muscle was then peeled downward to expose bony landmarks (**Figure 1E**). Intraoperative resection followed the tumor boundary with piecemeal resection performed to protect surrounding structures and ensure surgical safety. After the tumor resection procedure, dura closure with a water-tight suture and retention of the bone flap were performed (**Figure 1F**). Muscles were sutured with full anatomical reduction, and the scalp was sutured intradermally (**Figures 1G–I**).

## 3 Result

### 3.1 Clinical characteristics

Four patients (two men and two women) were included. The average age of the patients was 45.11 ± 11.62 years (range, 34–69 years). The cases involved one case of left foramen magnum meningioma, one case of right foramen magnum meningioma, one case of meningioma in the ventral medulla area, and one case of glioma of the ventral medulla.

### 3.2 Surgical outcome

Among all patients, there was a gross total resection and no recurrence after surgery. All cases exhibited satisfactory wound healing. Except for one case of left foramen magnum meningioma, which initially presented with dysphagia and pneumothorax but recovered within 6 months postoperatively, the other cases were without postoperative complications. All four cases were successful, and patients were followed up after discharge for 3–6 months, with an average of 4.12 ± 2.62 months. There were no new complications or recurrences in all cases.

### 3.3 Case illustration

#### 3.3.1 Case 1

**Figure 2:**
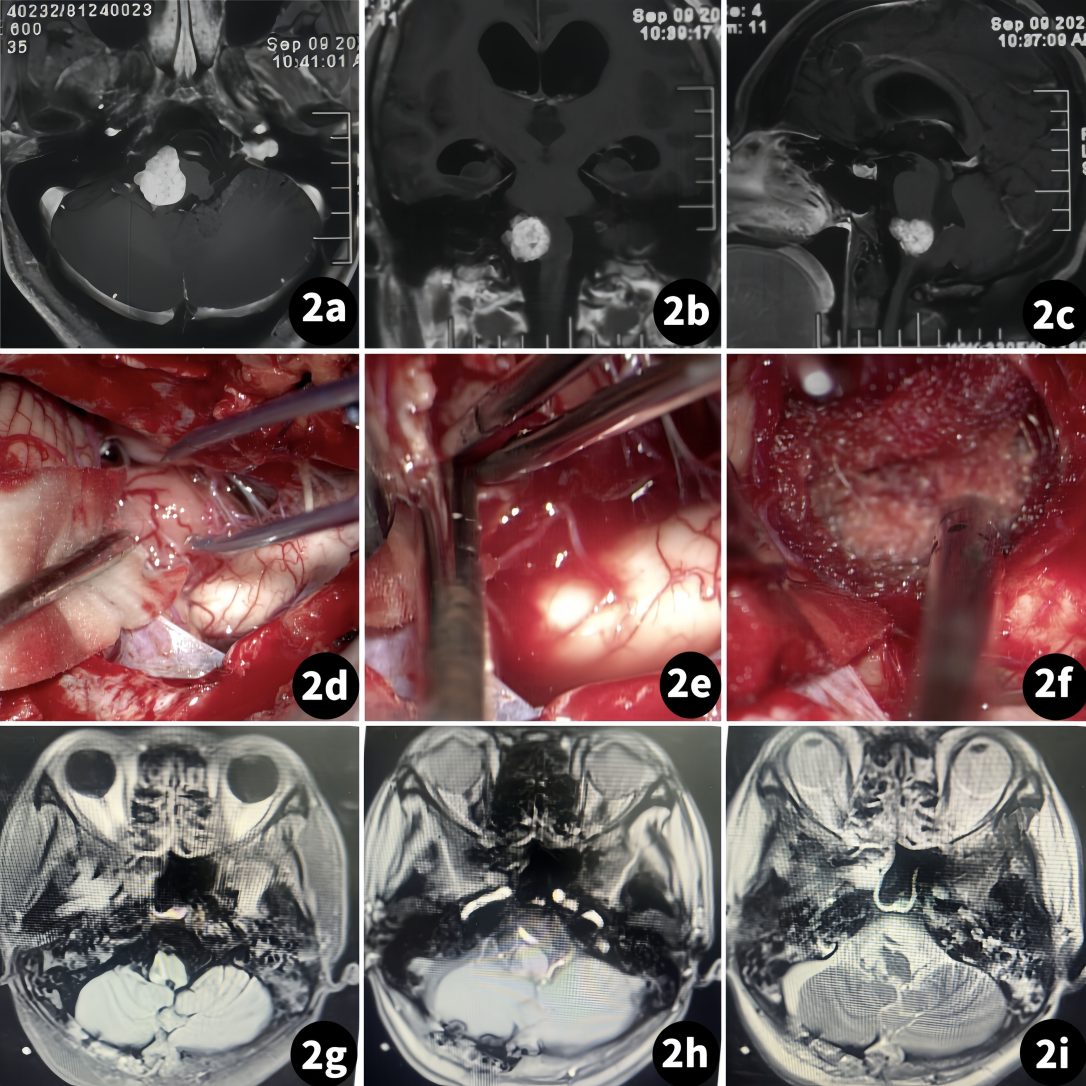
Surgical treatment of the right foramen magnum meningioma. (Case 1). **(A–C)** Preoperative MR scan; **(D–F)** Tumor resection procedure; **(G–I)** Postoperative MR scan.

The patient was a 35-year-old woman suffering from limb weakness for 2 months. A physical exam revealed that both upper and lower limbs had grade III muscle strength. An MR scan showed an occupation in the right foramen magnum area, with the medulla compressed by the tumor mass (**Figure 2**).

#### 3.3.2 Case 2

**Figure 3:**
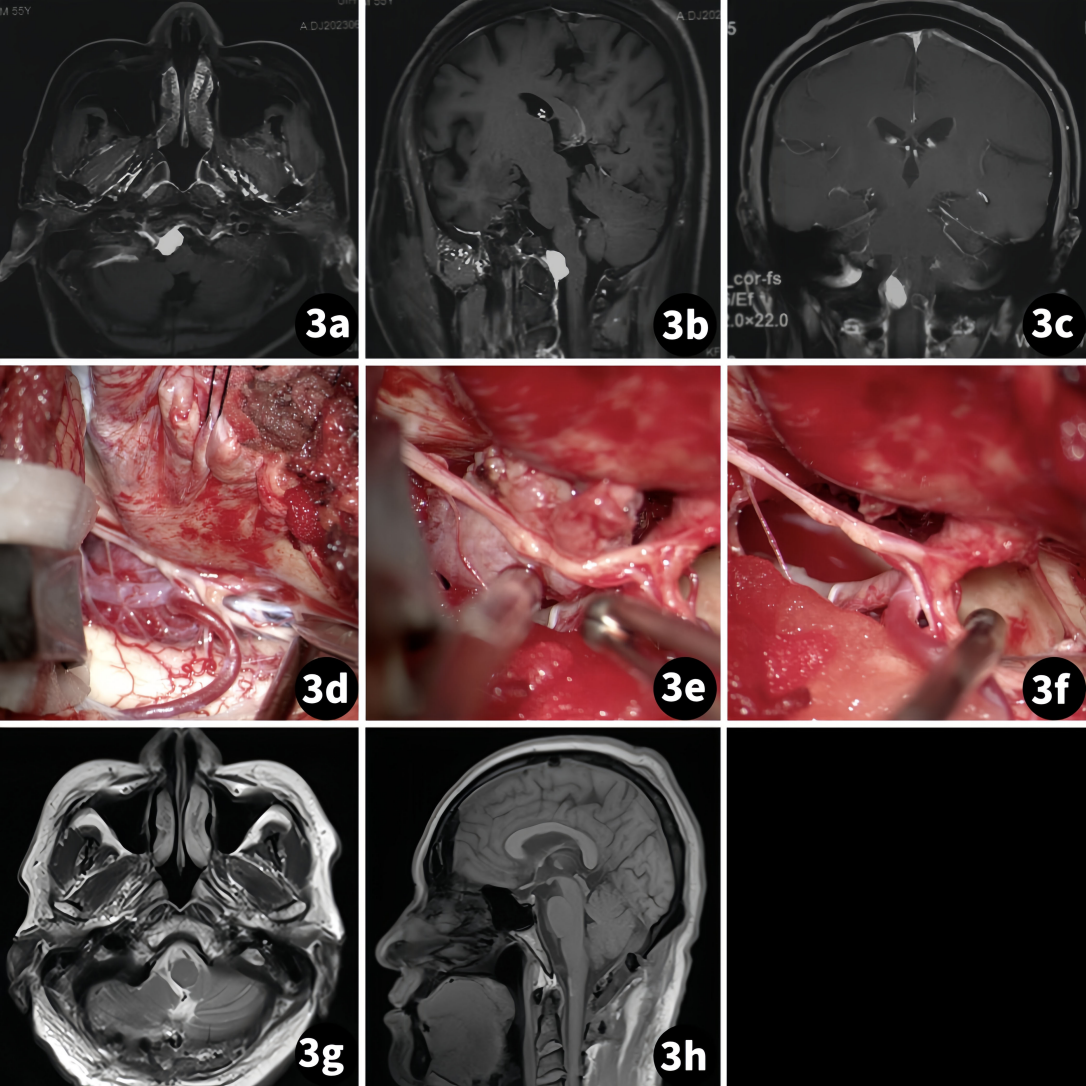
Surgical treatment of the right foramen magnum meningioma. (Case 2) **(A–C)** Preoperative MR scan; **(D–F)** Tumor resection procedure; **(G, H)** Postoperative MR scan.

The patient was a 55-year-old man suffering from intermittent headaches for more than 6 months. There was no neurological deficit found during physical examination. An MR scan showed a tumor was on the right side of the foramen magnum with homogeneous enhancement (**Figure 3**).

#### 3.3.3 Case 3

**Figure 4:**
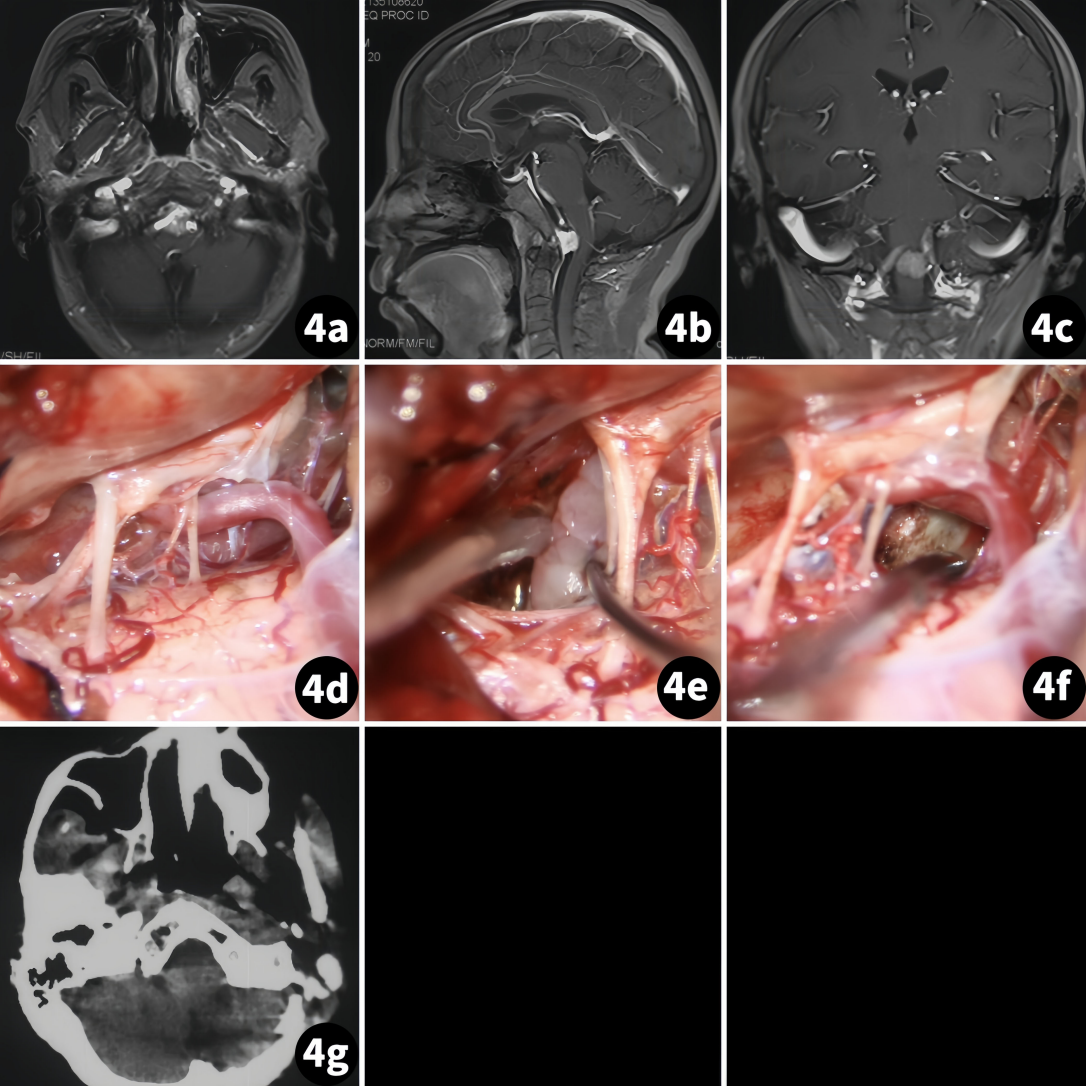
Surgical treatment of the foramen magnum meningioma in the ventral medulla. (Case 3) **(A–C)** Preoperative MR scan; **(D–F)** Tumor resection procedure; **(G)** Postoperative MR scan.

The patient was a 69-year-old woman suffering from dizziness accompanied by intermittent headaches for over 5 months. A physical exam revealed limitations in the fine motor skills of both upper limbs. An MR scan showed homogeneous enhancement of a tumor mass located on the ventral side of the medulla (**Figure 4**).

#### 3.3.4 Case 4

**Figure 5:**
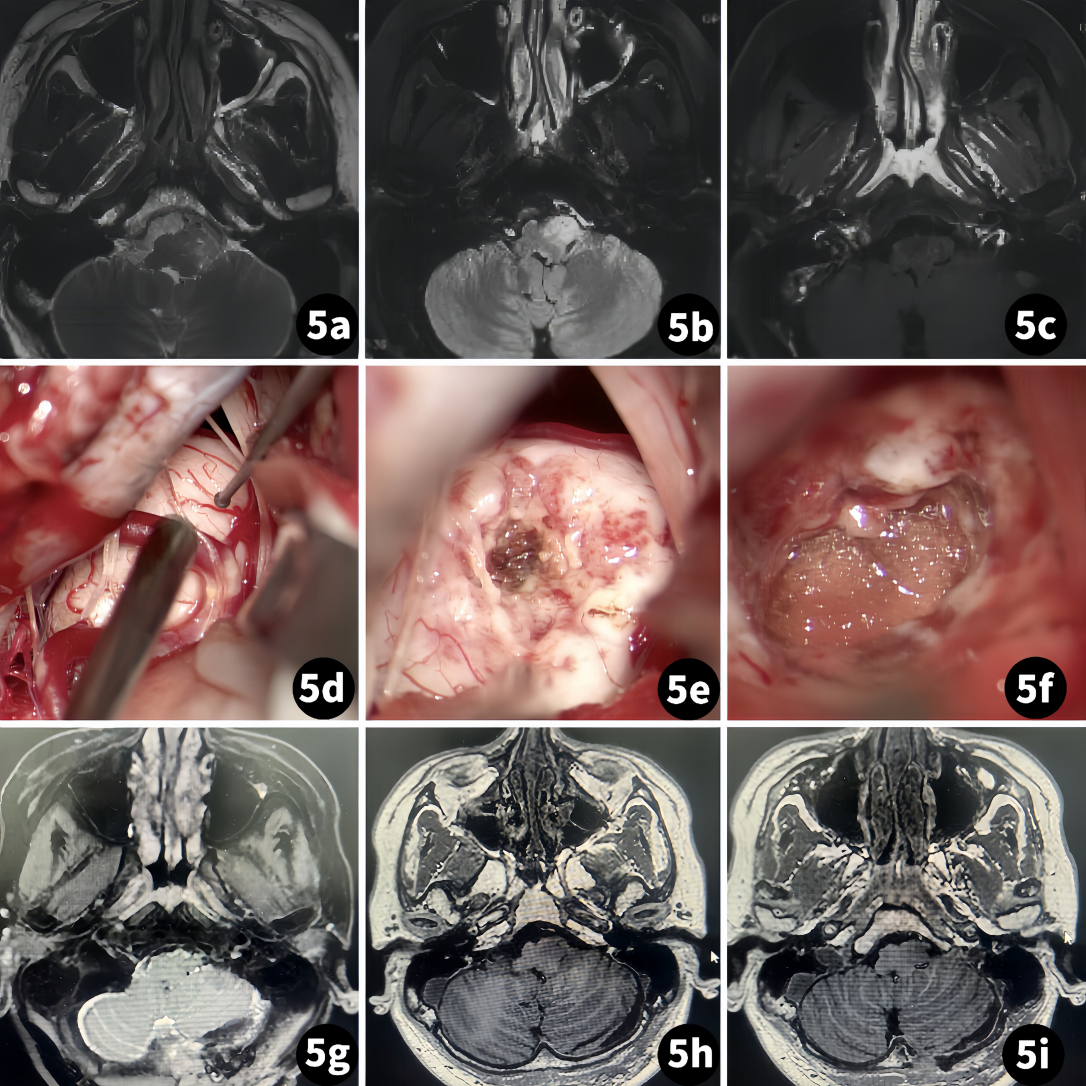
Surgical treatment of a glioma of the ventral medulla. (Case 4) (**A–C)** Preoperative MR scan; **(D–F)** Tumor resection procedure; **(G–I)** Postoperative MR scan.

The patient was a 34-year-old man. A routine examination found a mass in the left brainstem for 2 weeks. A physical exam revealed that both the left upper and lower limbs had grade III muscle strength. An MR scan showed no obvious enhancement, and the T2 sequence presented a relative high density (**Figure 5**).

## 4 Discussion

### 4.1 Disadvantages of traditional skin incision in a far-lateral approach application

A far-lateral approach is a common approach to dealing with lesions on the ventral side of the foramen magnum area and jugular foramen area (11, 12). Various surgical incisions using this approach have been reported in the literature, such as the “C”-shaped incision, the reverse “U”-shaped incision, the crutch-shaped incision, and the lateral retrooccipital “S”-shaped incision (13–17). These incisions often result in larger exposure to the subcutaneous area and greater trauma, leading to complications such as CSF leakage and scalp hydrops. Few researchers attribute these postoperative complications to muscle damage during craniectomy, failure of anatomical reduction, and extensive subcutaneous exposure (18–20). In clinical practice, we have encountered similar situations. Minimizing exposure and keeping the muscle intact for complete anatomical reduction may reduce these complications (**Table 2**).

**Table 2:** Comparison of different incisions: C-shaped, transverse-S, “reverse hockey stick,” and straight.

Incisions	Patients position	Skin incision	Muscle dissection	Advantages	Disadvantages
**C-shaped**	The lateral or park-bench position	Incision starts with 4 cm behind the mastoid tip/transverse process of C1, and the skin flap flushes with the line connecting the sigmoid sinus, the mastoid tip, and the transverse process of C1.	The sternocleidomastoid muscle was reflected detached from the insertion and reflected anteriorly with the flap.	Prevents CSF leakage and the formation of a pseudomeningocele.	Larger exposure to the subcutaneous area and greater trauma.
**Transverse-S**	The lateral or park-bench position	The incision starts at the retroauricular area below the asterion.	Subperiosteal detachment of the muscles in a single layer from the superior nuchal.	Provides enough space for muscle retraction and bone exposure.	Larger exposure to the subcutaneous area.
**“Reverse hockey stick”**	The lateral or park-bench position	A sigmoid or hockey stick-type incision behind the ear in a linear fashion centered over the C1 transverse process.	Blunt dissection of the neck muscles’ layers	Greater muscle layer exposure to the surgical area.	a. Muscles cannot be cut by passivative dissection; b. Increasing the distance between the surgeon and the surgical area.
**Straight**	3/4 prone	The incision edge is positioned below the hairline of the ear, with the lowest edge reaching the C4 spinous process and the highest edge extending 1 cm above the external occipital protuberance.	Muscles were sutured with a fully anatomical reduction.	Simplifies the craniectomy procedure and better preserved the integrity of muscle structure.	Lack of relative cases.

C1, cervical vertebra 1.

### 4.2 Advantages of oblique straight incision applied in a far-lateral approach

We use an oblique straight incision over the C4 spinous process and 1 cm above the level of the external occipital protuberance. The main advantage of this incision is the reduction of subcutaneous area exposure. Secondly, stretching the splenius capitis muscle to follow its fibers, not cut through it, ensured its integrity. Thirdly, the semispinalis capitis muscle was cut from the upper margin and turned to the lower edge, maximizing exposure to the suboccipital triangle. In all four cases, this method provided adequate exposure and fully met the requirements of the intracranial procedure. All muscles were fully anatomically reduced with a close suture. Ensuring a close suture of the dural membrane after surgery is still a crucial step (if unable to suture, dural substitute and fibril glue are needed). Additionally, bone wax seals the mastoid air chamber, closes the scalp suture, etc. (21–25).

### 4.3 Complications

Except for one case of a patient with a right foramen magnum meningioma with dysphagia who recovered after conservative treatment postoperatively (6 months) and pneumothorax postoperatively (4 h) after conservative and ventilator therapy, other cases were without postoperative complications. The pneumothorax was likely caused by a subclavian puncture during anesthesia. The postoperative dysphagia was attributed to the tight adhesion of the tumor to the glossopharyngeal nerve, making the dissection procedure difficult.

### 4.4 Limitation

Firstly, the small number of cases may have influenced our results. Secondly, some variation was inevitable when clinically applying this method. Therefore, more studies are needed to further validate our findings.

## 5 Conclusion

A far-lateral approach using an oblique straight incision can preserve muscle integrity and minimize subcutaneous exposure, allowing for complete anatomical reduction of muscles. This craniectomy method is simple and replicable, making it worthy of further clinical practice.

## Data availability statement

The original contributions presented in the study are included in the article/**Supplementary Material**. Further inquiries can be directed to the corresponding authors.

## Ethics statement

Written informed consent was obtained from the individual(s) for the publication of any potentially identifiable images or data included in this article.

## Author contributions

JB: Writing – review & editing. Z-hJ: Writing – original draft. PC: Conceptualization, Investigation, Methodology, Software, Writing – review & editing, Writing – original draft. YC: Conceptualization, Formal Analysis, Writing – review & editing. Y-mW: Data curation, Validation, Writing – review & editing. GC: Conceptualization, Formal Analysis, Writing – review & editing. X-rX: Conceptualization, Methodology, Project administration, Supervision, Validation, Writing – review & editing.
